# A novel technique for stabilising sacroiliac joint dislocation using spinal instrumentation: technical notes and clinical outcomes

**DOI:** 10.1007/s00068-021-01873-z

**Published:** 2022-01-13

**Authors:** Takahito Miyake, Kentaro Futamura, Tomonori Baba, Masayuki Hasegawa, Kanako Tsuihiji, Norihide Kanda, Yoshihiko Tsuchida, Atsuhiko Mogami, Osamu Obayashi, Shinji Ogura

**Affiliations:** 1 Advanced Critical Care Center, Gifu University Hospital, 1-1 Yanagido, Gifu-shi, Gifu, 501-1194 Japan; 2 Trauma Center, Shonan Kamakura General Hospital, 1370-1Okamoto, Kamakura-shi, Kanagawa, 247-8533 Japan; 3 Department of Orthopaedic Surgery, Juntendo University, 3-1-3 Hongo, Bunkyo-ku, Tokyo, 113-8431 Japan; 4 Department of Orthopaedic Surgery, Juntendo Shizuoka Hospital, 1129 Izunagaoka, Izunokuni, Shizuoka 410-2295 Japan

**Keywords:** Sacroiliac joint dislocation, Sacroiliac joint stabilisation, Sacroiliac joint crescent fracture–dislocation, Unstable pelvic ring fractures, Iliosacral disruption, Internal fixation

## Abstract

**Purpose:**

Currently, sacroiliac joint dislocations, including crescent fracture–dislocations, are treated using several techniques that have certain issues. We present the technical details and clinical outcomes of a new technique, anterior sacroiliac stabilisation (ASIS), performed using spinal instrumentation.

**Methods:**

ASIS is performed with the patient in a supine position via the ilioinguinal approach. The displacements are reduced and fixed by inserting cancellous screws from the sacral ala and iliac brim; the screw heads are bridged using a rod and locked. We performed a retrospective review of patients with iliosacral disruption who underwent ASIS between May 2012 and December 2020 at two medical facilities. The patients were assessed for age, sex, injury type, associated injuries, complications, functional outcome by evaluating the Majeed pelvic score after excluding the sexual intercourse score and fracture union.

**Results:**

We enrolled 11 patients (median age: 63 years). The median operative time was 195 min, median blood loss was 570 g, and eight patients (72.3%) required blood transfusion. The sacral and iliac screws had a diameter of 6.0–8.0 mm and 6.2–8.0 mm, and a length of 50–70 mm and 40–80 mm, respectively. Bone union was achieved with no marked loss of reduction in the median follow-up period of 12 months in all cases. The median Majeed score at the final follow-up was 85/96.

**Conclusion:**

ASIS is a rigid internal fixation method that provides angular stability. Despite invasiveness issues compared to iliosacral screw fixation, this method is easy to confirm and achieves precise reduction.

## Introduction

Sacroiliac joint dislocations, including crescent fracture–dislocations, are treated as unstable pelvic ring injuries. If these injuries are managed with persistent pelvic ring instabilities or deformities, functional disorders associated with leg length discrepancy, gait abnormalities, sitting problems, and low back pain could remain. Therefore, several reports have highlighted the need to achieve anatomical alignment while treating these injuries [1, 2].

Internal fixation methods for sacroiliac joint dislocation described in previous reports include iliosacral screws, anterior plates, and osteosynthesis using spinal instrumentation via the posterior approach [3].The most commonly used technique to stabilise pelvic ring injuries is iliosacral screw fixation. Since this technique is performed percutaneously, the lag screws can be inserted perpendicular to the iliosacral joints and compressed less invasively [4, 5]. Despite the advantages of this technique, reduction of the ilioscaral joint should be completed before insertion of the iliosacral screws, and the fixation process should be performed in all cases after completing reductions. Anterior plating could be a choice to reduce and fix the sacroiliac joints simultaneously. However, no implant provides adequate angular stability in general. Osteosynthesis using spinal instrumentation by the posterior approach may be a good option to achieve better stability; however, several difficulties involved in reducing the sacroiliac joint in a prone position have been reported [6]. Thus, there are advantages and disadvantages for each treatment strategy, and a standard fixation technique has not been established. An internal fixation method that is minimally invasive, easy to perform, and achieves precise reduction with rigid stability is required.

Therefore, one of the authors of this study developed an anterior sacroiliac stabilisation (ASIS) technique using spinal instrumentation as a new internal fixation method to address the problems associated with other methods, such as the difficulty in implant placement. This technique achieved precise reduction with adequate fixation stability. This study aimed to describe the detailed operation technique of ASIS and report the clinical outcomes of this surgery. The current study design was approved by the institutional review board and the ethical committee.

## Materials and methods

### Materials

A titanium alloy-based spinal instrumentation (USS II Ilio-Sacral, Modular System for Stable Fixation in the Sacrum and Ilium, Depuy Synthes Co., Zuchwil, Switzerland) was used. The sacral and iliac USS II dual-core cancellous polyaxial screws have an outer diameter of 6.2–8.0 mm and length of 30–80 mm (length is 65 mm only in a screw with an outer diameter of 6.2 mm). The inner diameters of the screws correspond to the cancellous and cortical bone thickness, which increase the pull-out strength. Rod-bridging screws have two diameters (5.0 and 6.0 mm). The instrument is equipped with a polyaxial 3-D head, sleeve, and nuts to connect the rod and screw, enabling the assembly of a side-loading polyaxial screw system, a combination of the screw head and sleeve that retains the rod as if it is wrapped. Thus, the force loaded on the nut is reduced, resulting in a firm construction. In addition, the rod can be easily set because the head has circumferential mobility of ± 25°. We utilised the implants of the EXPEDIUM VERSE spinal system (Depuy Synthes Co., Zuchwil, Switzerland) for the last four cases. Differences between the USS II Ilio-Sacral and EXPEDIUM VERSE spinal system are that in the latter, the screws are cannulated (Verse cannulated CFX screws) and have size variations. The rod has a fixed diameter of 5.5 cm, and it is stabilised by a top-loading system. Moreover, the most significant differences are in the structures and properties of the screw heads and set screws. In the EXPEDIUM VERSE spinal system, screw heads are available with favoured angles and can be selected from a maximum of 50° to a minimum of 20°, and the set screws, called the “correction keys”, can lock the angles of the heads. At this point, the rod slides in the screw heads, and compression is applied along the rod between the screws so that the implant can be stabilised using the locking properties. The unitised set screws can easily achieve the final locking if no compression is necessary.

### Patients

Of the patients with pelvic ring fractures who underwent surgery between May 2012 and December 2020 at two medical facilities, ASIS was performed in 11 patients for sacroiliac joint fracture–dislocation or sacroiliac joint dislocation.

### Preoperative set-up

For ASIS, the patient was placed in a supine position on a radiolucent operating table with access to a C-arm fluoroscopic unit under the table for anteroposterior, inlet, and outlet projections. The surgical field and the lower limb on the affected side were disinfected and draped to prepare for manual or direct traction during surgery.

### Approach

A skin incision of approximately 12 cm was made along the iliac crest starting at the medial point of the anterior superior iliac spine. This is the first window in the ilioinguinal approach. The external oblique muscle was elevated off and over the iliac crest. After entering the iliac fossa, the iliacus muscle was elevated off the inner table using a Cobb elevator, and the psoas muscle was elevated over the iliac brim. Then, the abdominal and pelvic contents were retracted. Feeding vessels to the iliopsoas muscles located 1–2 cm lateral to the anterior line of the sacroiliac joint, which is at the iliac fossa, were ligated. Bleeding was controlled using bone wax if these vessels were injured and occluded by thrombosis.

The sacroiliac joint is covered by the anterior sacroiliac ligament and appears to bulge, making it easy to identify. The injured ligaments were excised to confirm the reduction of the sacroiliac joint. The methods achieved better visualisation of the area extending from the sacroiliac joint or iliac fossa to the proximal side of the arcuate line. First, a large retractor was inserted at the medial side of the sacroiliac joint, and then the iliacus was retracted medially to gain direct visualisation of the sacral ala after carefully securing the L5 nerve root. Second, an intestinal spatula was inserted into the proximal side of the arcuate line, locating the lesser pelvis just above the greater sciatic notch, with the bone serving as a fulcrum, after retracting the skin medially and distally.

### Reduction and fixation

**Fig. 1:**
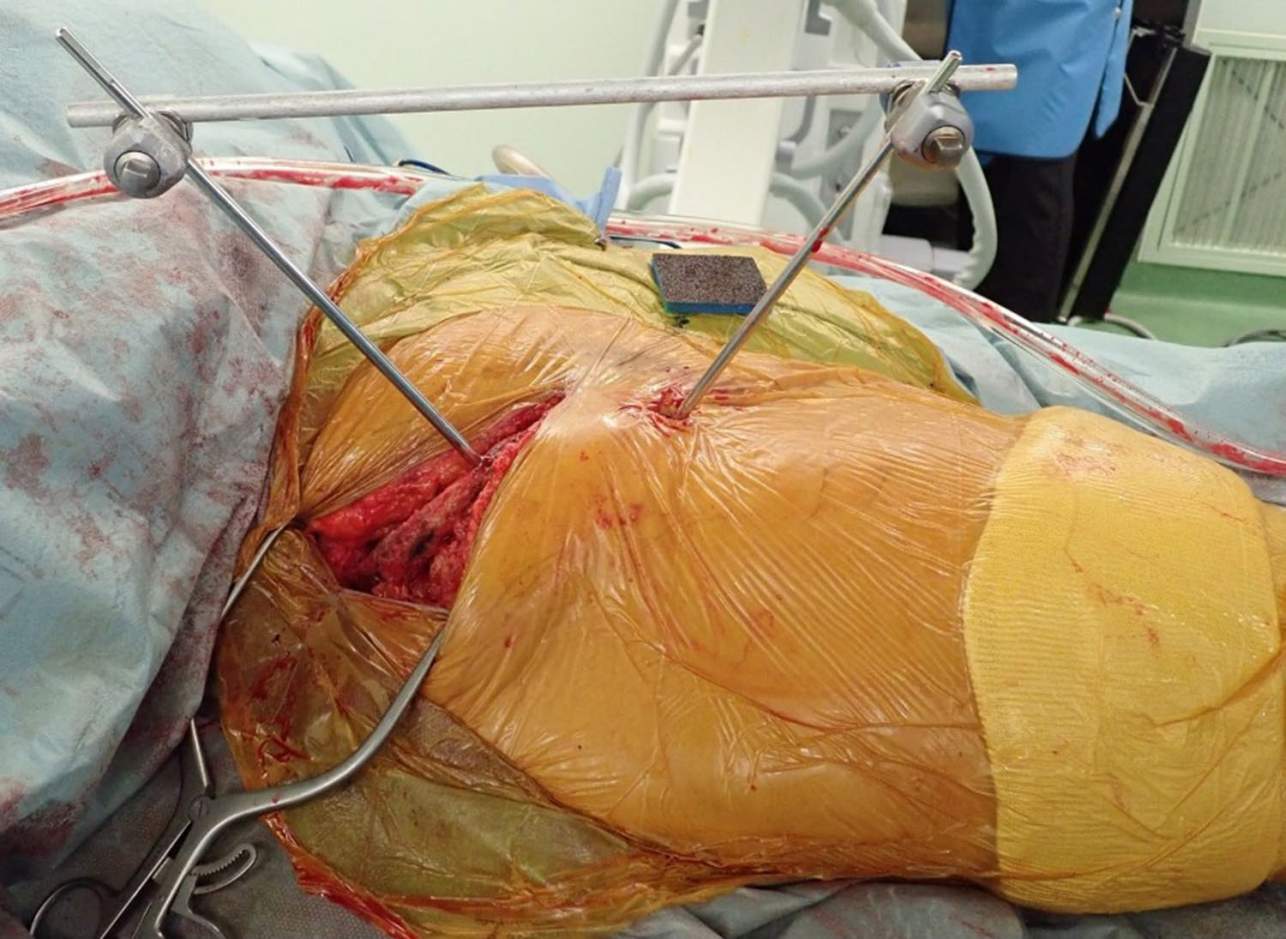
Reduction. Schanz pins of 5 mm diameter were inserted along the high route (black asterisk) and low route (black star) each. Bridging between the pins was performed using the Hoffmann II External Fixation System (Stryker, Kalamazoo, MI, USA), and reduction of the iliosacral joints was achieved by controlling the frames

Reduction of the dislocated ilium was achieved by a combination of several techniques. A Schanz pin with a diameter of 5 mm was inserted into the iliac crest using the joy-stick manoeuvre (Fig. 1). A ball-point spike pusher to push the ilium and distraction of the lower leg are the choices for reducing rotational and vertical displacements.

**Fig. 2:**
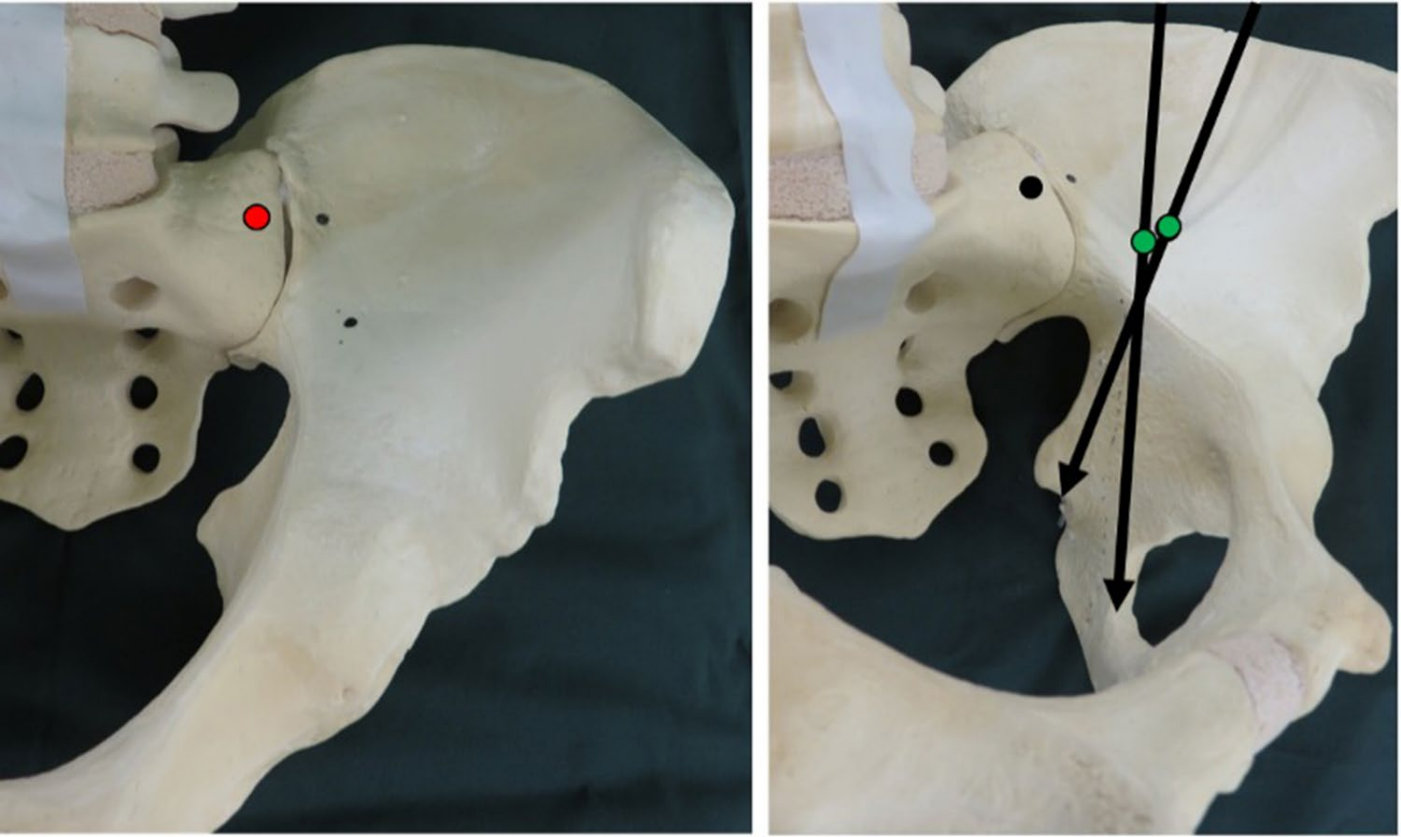
Entry points of sacral and iliac screws. **a** Entry point of the sacral screw (red circle) is at the centre of the sacral ala slope. **b** Entry point of iliac screw is 2 cm lateral and 1 cm distal to the anterior point of the sacroiliac joint (green circle). Goal of the screw is to reach the ischial spine or ischial tuberosity (indicated by black arrows)

**Fig. 3:**
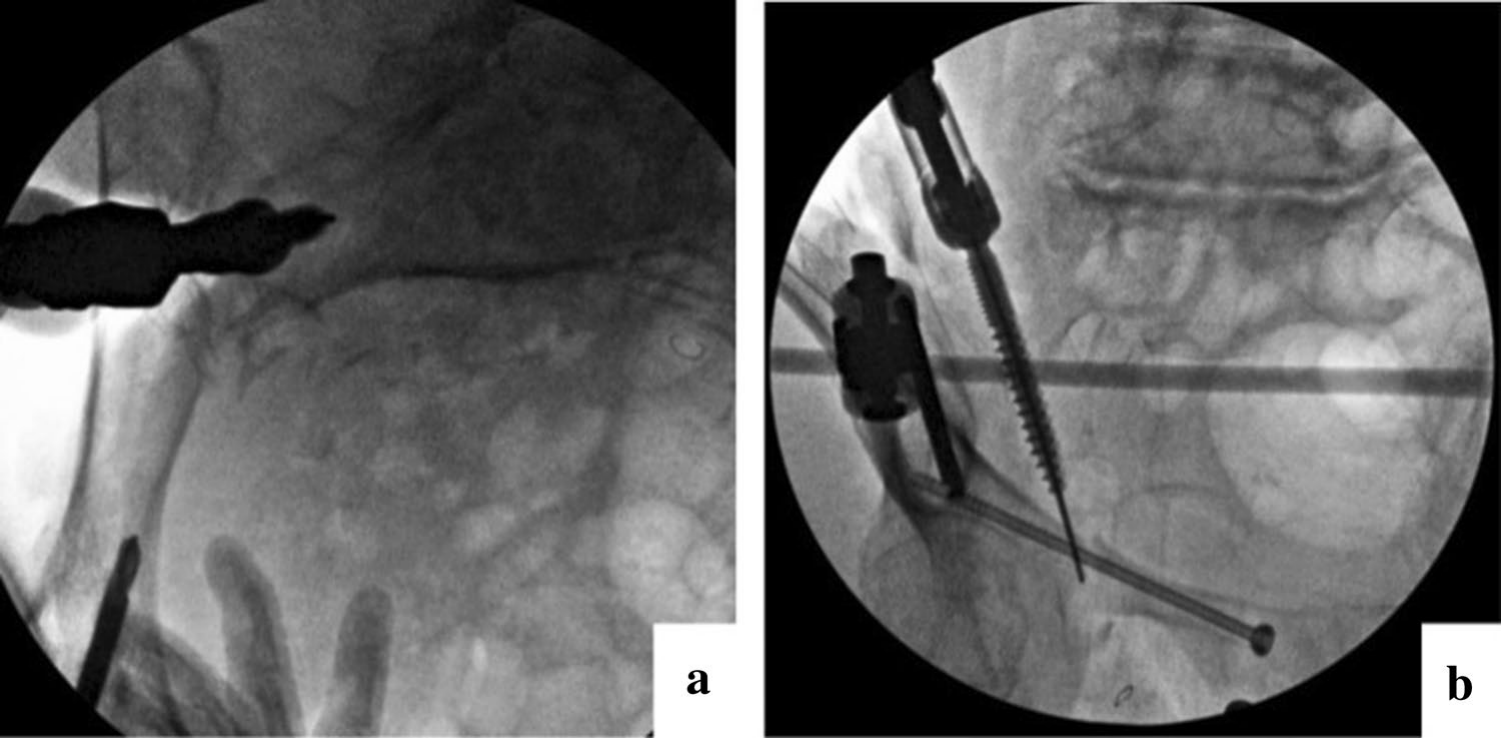
**a** Sacral screw insertion in the inlet oblique view. After the inlet view is checked, the C-arm is tilted to the injured side to display the sacroiliac joint more clearly. It is important to confirm that the sacral screw is inside the bones and does not pass through the sacroiliac joint (inserted screw is seen in the figure, not a probe). **b** Sacral screw insertion in the outlet oblique view. After the outlet view is checked, the C-arm is tilted to the injured side to display the sacroiliac joint more clearly. It is important to confirm that the sacral screw does not pass through the sacral foramina or sacroiliac joint (inserted screw is seen in the figure, not a probe)

The fifth lumbar nerve root was securely retracted, and the entry point of the sacral screw was made at the medial side of the sacroiliac joint and the centre of the lateral part of the sacrum. Insertion points for iliac and sacral screws are described in Fig. 2a, b. Subsequently, a probe was inserted in an appropriate direction through the entry point. While inserting the probe, an inlet oblique view was used to confirm that the probe tip did not to go outside the sacrum and passed through the sacroiliac joint (Fig. 3a). The probe was maintained parallel to the sacroiliac joint and on the lateral side of the sacral foramina in the anteroposterior and outlet views. An outlet oblique view was obtained by tilting the C-arm to display the sacroiliac joint more clearly, which was more useful in deciding the precise insertion route (Fig. 3b). When the probe was advanced into the sacrum, the surgeon ensured the ideal insertion direction by feeling the long screw using the hand. The screw insertion route was then confirmed as safely secure using a pedicle sounder, and a screw with a 70 mm diameter was inserted.

**Fig. 4:**
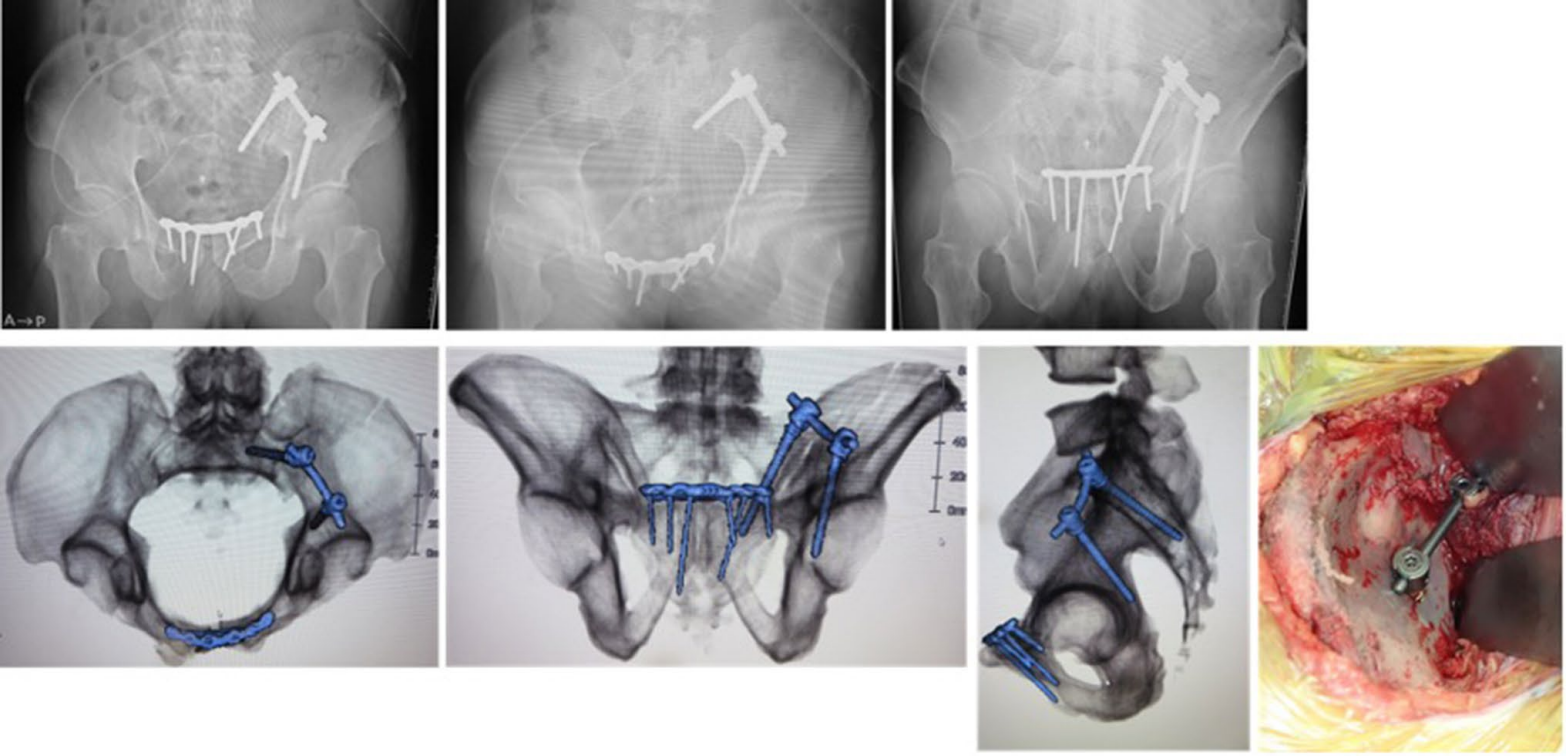
Postoperative radiographic images and a photograph of the operation. **a** Postoperative anteroposterior view radiograph. **b** Postoperative inlet view radiograph. **c** Postoperative outlet view radiograph. **d** Postoperative reconstructed inlet view 3D-computed tomography (CT) image. Screw is not outside the bone or in the sacroiliac joint. **e** Postoperative reconstructed outlet view 3D-CT image. Long screw has been inserted without passing through the sacral foramina or sacroiliac joint. **f** Postoperative reconstructed lateral view 3D-CT image. Long screw (70 mm) has been inserted in the sacrum. **g** A photograph of anterior sacroiliac stabilisation (different case than **a**–**f**). Surgical site is widely visualised, and the screws are bridged using a rod above the sacroiliac joint

Next, the insertion point and direction of the iliac screws were determined. The entry point was 2 cm lateral and 1 cm distal to the anterior point of the sacroiliac joint, and the goal of the screw was to reach the ischial spine or ischial tuberosity, following a direction similar to that of the posterior column screw for acetabular fractures (Fig. 2b). The screw was inserted carefully to avoid entry into the hip joint. The insertion process was the same as that performed for the sacral screw. The screw heads were bridged using a rod. In the USS II Ilio-Sacral system, rod bending and connection using a side-loading polyaxial system are needed. In the EXPEDIUM VERSE spinal system, rod connection is easier because of the top-loading polyaxial system, which allows attachment of a rod on the groove of the screw head (Fig. 4).

**Fig. 5:**
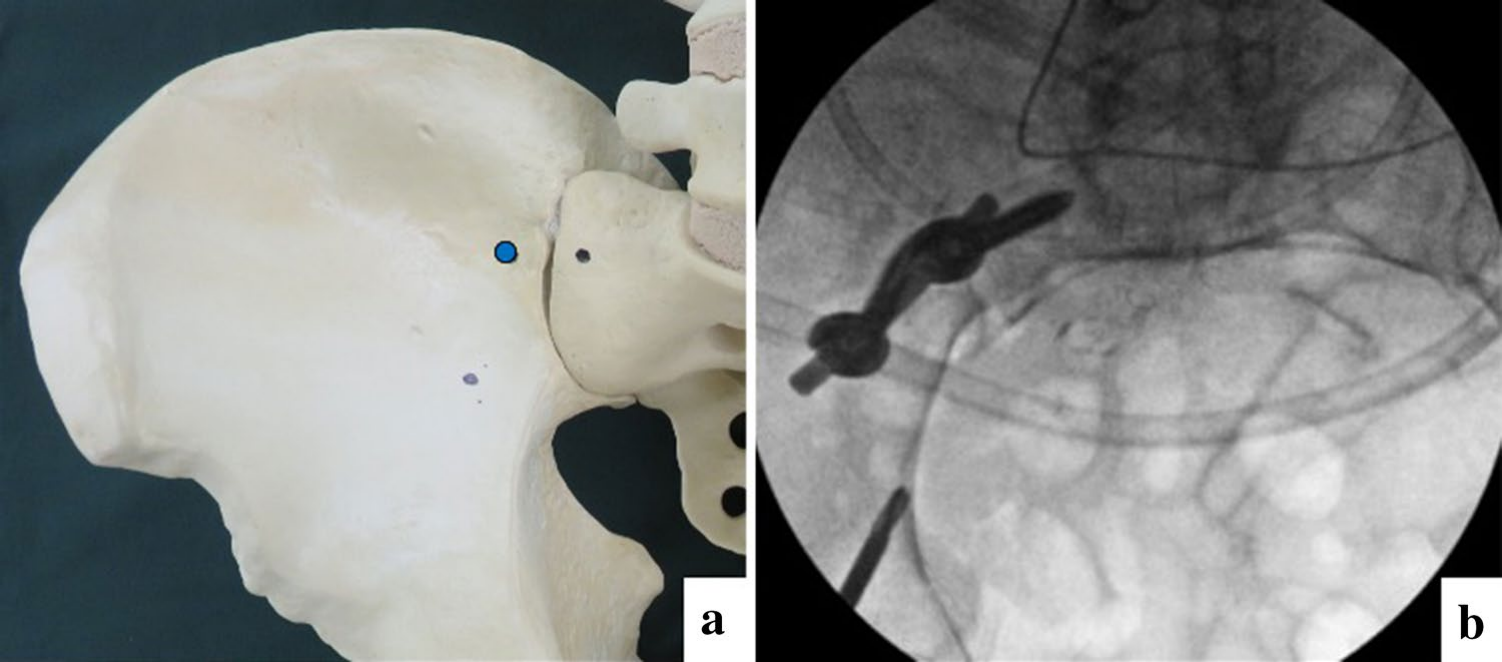
Compression of the sacroiliac joint by anterior sacroiliac stabilisation. The direction of the iliac screw should be along the sacral screw (blue circle) and parallel to the sacroiliac joint in the intraoperative inlet view, heading toward the posterior superior iliac spine (PSIS). If a strong reduction force is needed for fixing sacroiliac joint dissociation, the iliac screws should be in the direction of PSIS and should not be inserted into the posterior column

If a reduction force from the implants is needed to fix the sacroiliac joint dislocation, the direction of the iliac screw should be along the sacral screw and parallel to the sacroiliac joint, heading toward the posterior superior iliac spine, and the screw should be as long as possible. The head of the sacral screw and the rod were attached first, followed by compression between the screws along the rod with a compressor, and finally, the iliac screw and rod were locked (Fig. 5).

## Results

### Patient characteristics

The median age was 63 years (interquartile range [IQR]: 26–74 years), and five and six patients were men and women, respectively. The cause of injury was a fall from a height in two patients, traffic accident in six, compression by a heavy item in one, and fall from a standing position in two. The fracture type was AO/OTA class 61B1.1 in two patients, B2.2 in five, B2.3 in two, and C1.2 in two.

### Clinical results

All procedures were performed as planned after confirming that bleeding had stopped based on the vital signs and laboratory data. The median operative time was 195 min (IQR: 163–239 min), the median blood loss was 570 g (IQR: 381–945 g), 8 patients (72.3%) required blood transfusion, and the median volume of blood transfusion was 560 mL of red cell concentrate (IQR: 0–560 mL) and 0 cm^3^ of fresh frozen plasma (IQR, 0–480 mL). Inserted sacral and iliac screws were of diameter 6.0–8.0 mm and 6.2–8.0 mm and length 50–70 mm and 40–80 mm, respectively. Two patients underwent other fracture operations (lower leg fracture, 1; distal radius fracture, 1) along with ASIS. Clinical courses were surveyed for all patients. The median follow-up period was 12 months (IQR: 9–44 months), during which bone union was achieved, and no marked loss of reduction occurred in all cases. Deep infection occurred in one patient and was treated with lavage and debridement. The Majeed pelvic score [7] is a patient-reported outcome obtained by evaluating five areas: pain (30 points), work (20 points), sitting (10 points), sexual intercourse (4 points), and standing (36 points). At the final follow-up, the median Majeed score was 85 (IQR: 79–90) out of 96, excluding the sexual intercourse score. Patient demographic data and details of the operations and outcomes are summarised in Table 1.

**Table 1:** Patients’ demographic data and details of the operations, outcomes

Patinet number	Gender (male/female)	Age	ISS	Associated injuries	AO/OTA classification	Blood loss (g)	Transfusion	Operation time (min)	Implant	Sacral screw diameter-length (mm)		Note	Complication/reoperation related to the pelvic fracture	Final follow-up duration (month)	Majeed pelvic score except for sexual intercourse (96 points)
										Sacrum	Ilium				
1	F	63	9	Lt. humeral fracture	61B1.1	1990	RCC 2800 ml, FFP 4500 ml, PC 200 ml	229	USS-II	7.0–70	7.0–55	–	–	48	79
2	F	67	9	Bil. fibular fracture, Rt. Tibial fracture	61C1.2	660	RCC 560 ml	195	USS-II	7.0–55	7.0–50	–	–	45	96
3	M	26	16	Rt. hip dislocation	61C1.2	3500	RCC 560 ml, FFP 480 ml, intraoperative blood salvage 1400 ml	360	USS-II	7.0–65	7.0–60	–	–	44	85
4	F	53	9	Rt. tibial open fracture, Lt. tibial fracture	61B1.1	381	RCC 560 ml	239	USS-II	8.0–60	6.2–55	ORIF for tibia fracture in the same operation	–	12	79
5	F	19	13	Mandibular fracture	61B2.2	570	–	190	USS-II	7.0–55	7.0–40	–	–	24	87
6	M	56	9	–	61B2.3	890	Intraoperative blood salvage 100 ml	362	USS-II	7.0–70	7.0–60	Anterior pelvic plating in the same operation	–	9	90
7	M	64	9	Lt. distal radial fracture	61B2.2	45	–	203	USS-II	7.0–70	8.0–80	ORIF for distal radial fracture in the same operation	–	5	85
8	F	94	9	–	61B2.2	400	RCC 560 ml	146	Expedium	7.0–50	8.0–65	–	–	12	74
9	M	80	9	–	61B2.2	945	RCC 840 ml, FFP 720 ml	163	Expedium	6.0–50 × 2	8.0–60 × 2	–	Deep infection	14	67
10	F	74	17	Liver injury, multiple rib fractures	61B2.2	495	Intraoperative blood salvage 120 ml	158	Expedium	7.0–60	7.0–50	–	–	9	96
11	M	20	17	C2 fracture, multiple rib fractures	61B2.3	280	–	165	Expedium	8.0–40	8.0–45	Anterior pelvic plating in the same operation	–	7	90

*Bil* bilateral, *FFP* fresh frozen plasma, *Lt* left, *ORIF* open reduction and internal fixation, *RCC* red cell concentrate, *Rt* right, *PC* platelet concentrate

## Discussion

The greatest advantages of ASIS include the ability to insert thick and long screws in the sacrum and ilium and for bridging and locking these screws with angular stability. Although attention should be paid to avoid injury to the fifth lumbar [8] or the first and second sacral nerve roots while inserting the sacral screw, a strong fixation force can be expected because of the thick sacral screw. Additionally, if the iliac screw is inserted in the same way as a posterior column screw without passage through the hip joint, the fixation force can be further strengthened using a longer screw [9]. If a reduction force from the implant is needed to fix the sacroiliac joint dissociation, the direction of the iliac screw should be along the sacral screw and parallel to the sacroiliac joint, heading toward the posterior superior iliac spine, and the screw should be as long as possible. This procedure leads to easier compression between the screws, and the rod and screws can be locked immediately after completing the reduction. If concerns about fixation stability remain, another ASIS can be added (Case 9). There should be a gap for the second screw on the sacrum in these cases.

Thus, the surgical indications of ASIS are pelvic ring disruption with sacroiliac joint fracture–dislocations or sacroiliac dislocations which 1) need reduction from the anterior approach, 2) render it difficult to find the safety corridor for iliosascral screws fixation, and 3) need compression between dislocations.

The fixation stability of ASIS was not compared with that of other techniques; however, there was no loss of reduction or nonunion in our cases.

Currently, the generally accepted fixation technique for sacroiliac joint dislocations or sacroiliac fracture–dislocations is iliosacral screw fixation. The advantages of ASIS over iliosascral screw fixation are angular stability and variations in insertion points and directions of the screws, which allows changes in the installation of implants depending on fracture patterns or required fixation force. Moreover, it is difficult to insert iliosacral screws in some cases due to sacral dysmorphism. Common complications of iliosacral screw fixation are neurologic or vascular injuries associated with malposition of screws, which have a reported incidence of 2–15% [10, 11]. Although there have been reports that complications of this method can be overcome using a navigation based screw placement [12–14], even this method has several problems such as the learning curve [15] and a considerable financial investment for a fully equipped surgery room, lead-protection, and a reinforced floor and carbon table [16]. ASIS has certain advantages over other anterior techniques involving conventional plating, including the multi-angular stabilisation of dislocations.

The disadvantage of ASIS over iliosacral screw fixation is that it involves invasiveness of soft tissues and operative blood loss. In this study, there was one case of deep infection requiring debridement, the median operative time was 195 min, the median blood loss was 570 g, and eight patients (72.3%) required blood transfusion. These data indicate that ASIS can be inferior in terms of invasiveness when compared with iliosacral screw fixation [17, 18].

To our knowledge, this is the first report of internal fixation of the sacroiliac joint using spinal instruments via an anterior approach. Abumi et al. reported a fixation method for sacroiliac joint dislocations [3], wherein reduction and internal fixation for pelvic ring fractures was performed using spinal instrumentation via a posterior approach. In methods with a posterior approach, it is difficult to confirm effective reduction during the surgery because of the prone position of the patient [6]. ASIS has advantages over those methods in terms of a more effective reduction and confirmation of the reduction in the supine position.

This study has several limitations. It included only 11 cases, which is a small sample size, and combined several fracture types in the analysis. Further studies with more clinical cases are needed to verify the fixation stability in each fracture type. Ideally, a cadaveric study is warranted to confirm the rigidity of implants, breaking strength, and displacement of fractures biomechanically. Furthermore, this study did not compare the other fixation techniques: iliosacral screw or other techniques with the anterior approach. Further study is required to compare these techniques and establish the guidelines of the treatment indications for ASIS.

## Conclusion

In conclusion, ASIS is a rigid internal fixation method using thick and long screws inserted into the sacrum and ilium and bridged using a rod. It is a simple method that provides angular stability. Despite invasiveness issues, it is an effective method used to fix the crescent fracture and other dislocations of the sacroiliac joint.

## Data Availability

The data that supports the findings of this study are available from the corresponding author, TM, upon reasonable request.
